# Lesson From Practice: Not Gone, Should Not Be Forgotten: Recognising PIMS‐TS Cases in a Post‐Pandemic World

**DOI:** 10.5694/mja2.70181

**Published:** 2026-04-26

**Authors:** Rana Sawires, Anneke Engwerda, Mehyar Khair Baik, Philip N. Britton, David P. Burgner

**Affiliations:** ^1^ Monash University Melbourne Victoria Australia; ^2^ Murdoch Children's Research Institute Melbourne Victoria Australia; ^3^ Werribee Mercy Hospital Melbourne Victoria Australia; ^4^ Paediatric Active Enhanced Disease Surveillance Network, National Centre for Immunisation Research and Surveillance Sydney New South Wales Australia; ^5^ University of Sydney Sydney New South Wales Australia; ^6^ Children's Hospital, Westmead Sydney New South Wales Australia; ^7^ Royal Children's Hospital Melbourne Victoria Australia

**Keywords:** COVID‐19, PIMS‐TS, paediatric infectious diseases

## Abstract

We describe the clinical presentation, laboratory findings and complications of severe paediatric inflammatory multisystem syndrome temporally associated with severe acute respiratory syndrome coronavirus 2 (PIMS‐TS), also known as multisystem inflammatory syndrome in children in a 16‐year‐old female diagnosed in June 2025. This case highlights the importance of prompt diagnosis of PIMS‐TS in the post‐coronavirus disease 2019 pandemic era to ensure timely and appropriate management.

## 1 Clinical Record

A previously well 16‐year‐old female of mixed white–Thai ethnicity presented to an outer‐metropolitan emergency department after an unwitnessed syncopal episode, preceded by 1 week of fever, abdominal pain, vomiting and diarrhoea. She had no recent sick or coronavirus disease 2019 (COVID‐19) contacts and was fully immunised as per Australian guidelines. She was febrile and hypotensive on arrival and unresponsive to fluid resuscitation. She had bilateral conjunctival injection, without localising infectious focus. Initial investigations showed leucocytosis (white cell count, 17.8 × 10^9^/L; reference interval [RI], 4.0–11.0 × 10^9^/L), neutrophilia (12.3 × 10^9^/L; RI, 2.0–8.0 × 10^9^/L) and elevated C‐reactive protein (226 mg/L; RI, < 10 mg/L). Lymphocyte count was 1.2 × 10^9^/L (RI, 1.0–4.0 × 10^9^/L) and platelets initially 113 × 10^9^/L (RI, 150–450 × 10^9^/L, normalising by day 3 of admission). Urine and blood cultures were sterile, and respiratory multiplex panels (including severe acute respiratory syndrome coronavirus 2 [SARS‐CoV‐2] polymerase chain reaction [PCR] test) were negative. She had multiorgan dysfunction, including acute kidney injury (creatinine 162 μmol/L; RI, 40–80 μmol/L), hepatic transaminitis (γ‐glutamyl transferase, 121 U/L [RI, 0–20 U/L]; alanine aminotransferase, 46 U/L [RI, < 35 U/L]) and haemolysis. She required inotropic support, ceftriaxone and transfer to a tertiary paediatric intensive care unit (PICU).

In PICU, she received inotropes, broad‐spectrum antibiotics (clindamycin, flucloxacillin and ceftriaxone) and 1 g/kg intravenous immunoglobulin (Ig) for presumed toxic shock syndrome. She was weaned off inotropes within 36 h and defervesced and was transferred to the referring hospital for ongoing antibiotics.

**FIGURE 1:**
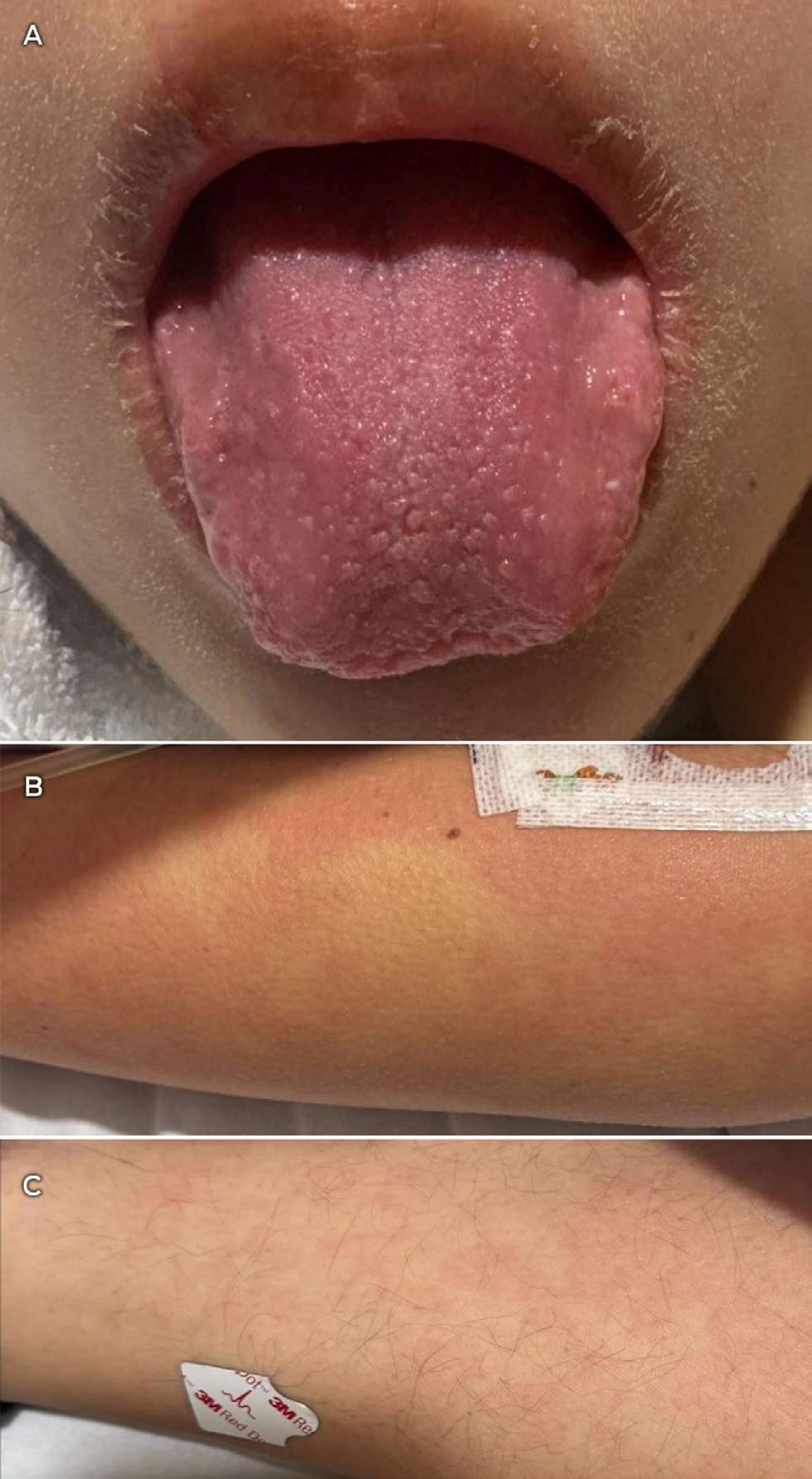
(A) Strawberry tongue, and (B, C) migratory maculopapular rash.

Two days after initial presentation, she deteriorated with hypotension and recrudescent fevers. She developed new signs including a migratory maculopapular rash and strawberry tongue (Figure 1). Investigations for a Kawasaki disease‐like syndrome were initiated, including transthoracic echocardiography (TTE), cardiac markers and anti‐spike and anti‐nucleocapsid SARS‐CoV‐2 serology.

In consultation with Paediatric Infectious Diseases, a diagnosis of probable PIMS‐TS was made. She was treated with further intravenous Ig (2 g/kg), intravenous methylprednisolone and low‐dose aspirin (5 mg/kg daily). Cardiac investigations demonstrated biochemical and structural dysfunction, with elevated troponins (peak, 49 ng/L; RI, < 11 ng/L) and right coronary artery dilatation (4.0 mm internal diameter) on TTE.

She improved over several days, with resolution of fever, hypotension, rash and conjunctival injection. Notably, she tested negative for SARS‐CoV‐2 on serial respiratory PCR panels. Her initial SARS‐CoV‐2 serology was positive for anti‐spike antibodies but negative for anti‐nucleocapsid IgG; a repeat sample 3 days later was positive against both antigens.

She was discharged after 11 days with ongoing low‐dose aspirin for thromboprophylaxis and a weaning steroid regimen. A repeat TTE after 6 weeks demonstrated no cardiac or coronary artery abnormalities.

## 2 Discussion

PIMS‐TS emerged during the COVID‐19 pandemic as a post‐infectious hyperinflammatory syndrome occurring in a minority of children 2–6 weeks after SARS‐CoV‐2 infection [1, 2]. Australian surveillance has identified 179 confirmed, 11 probable and 26 possible PIMS‐TS cases in Australia (as of 19 March 2026). PIMS‐TS shares features with Kawasaki disease, but is a distinct condition clinically and immunologically [2]. Three clinical phenotypes have been described, with abdominal symptoms more evident in older children [3]. Severe cases present with shock due to myocardial dysfunction. Evidence of recent SARS‐CoV‐2 infection is a key diagnostic criterion and may include positive serology, respiratory PCR or rapid antigen testing, or a confirmed close contact 2–6 weeks before onset [2]. Children more often have asymptomatic COVID‐19 compared with adults, yet post‐acute sequelae of COVID‐19, including PIMS‐TS, remain common. In this case, prior positive anti‐nucleocapsid IgG reflected recent infection, while anti‐spike IgG may reflect infection or vaccination [4].

PIMS‐TS incidence has declined with SARS‐CoV‐2 strain evolution and COVID‐19 vaccination [5, 6]. Australian surveillance confirms this decline but demonstrates that PIMS‐TS has not disappeared, as anecdotally reported in other countries. In Australia, confirmed cases fell from 132 in 2022 to 24 in 2023, 19 in 2024, 4 in 2025 (excluding the described case), and no cases until March 2026 [7], despite a rise in cases of Kawasaki disease, suggesting diagnostic misclassification [8]. This decline could further be attributable to challenges in meeting laboratory diagnostic criteria amid reduced COVID‐19 testing, given the high cost and perceived limited utility of SARS‐CoV‐2 serology in the era of endemic COVID‐19.

With increased recognition, prompt treatment, reduced severity coincident with evolving variants and partial protection from vaccination and/or previous infection, the mortality and morbidity of PIMS‐TS is lower than first described early in the COVID‐19 pandemic [9]. In contrast to Kawasaki disease, acute coronary artery changes in PIMS‐TS, which occur in 14%–26% of cases [2, 3], resolve rapidly, with less evidence of long‐term cardiac morbidity [10]. Notwithstanding, PIMS‐TS results in significant short‐term morbidity, including intensive care unit admission, prolonged hospitalisation, need for extended follow‐up and adverse emotional, psychosocial and educational impacts on children and caregivers [9, 10]. Early recognition of PIMS‐TS by frontline healthcare providers is essential for timely care for patients and families.

3

**Lessons From Practice**

- Paediatric inflammatory multisystem syndrome temporally associated with severe acute respiratory syndrome coronavirus 2 (SARS‐CoV‐2 [PIMS‐TS]) infection remains a clinically important condition with considerable morbidity.
- Recognition of PIMS‐TS enables timely, targeted and holistic patient care.
- Diagnosis is challenging, given the high rate of asymptomatic coronavirus disease 2019 (COVID‐19) in children and the evolving landscape of SARS‐CoV‐2 as an endemic virus.
- Persistent fever refractory to antibiotic therapy in the older child should raise concern for PIMS‐TS, and review of the history of presenting complaint, repeat examination and additional work‐up in this context can lead to timely treatment.

## Author Contributions

**Rana Sawires:** conceptualisation, data curation, visualisation, writing (original draft), writing (review and editing). **Anneke Engwerda:** data curation, supervision, writing (review and editing). **Mehyar Khair Baik:** writing (review and editing). **Philip N. Britton:** supervision, writing (review and editing). **David P. Burgner:** supervision, writing (review and editing).

## Funding

The authors have nothing to report.

## Disclosure

Not commissioned; externally peer reviewed.

## Consent

The patient's parent provided written consent for the publication.

## Conflicts of Interest

The authors declare no conflicts of interest.

## Data Availability

This article includes no original data.
